# There and back again: a cell biologist’s journey from organelles to molecules

**DOI:** 10.1515/hsz-2025-0185

**Published:** 2025-12-18

**Authors:** Emma J. Fenech, Yury S. Bykov

**Affiliations:** Center for Biochemistry and Center for Molecular Medicine Cologne, Faculty of Medicine, University of Cologne, D-50931 Cologne, Germany; Quantitative Cell Biology, 26562 RPTU Kaiserslautern-Landau, D-67663 Kaiserslautern, Germany

**Keywords:** organelles, protein complexes, organelle subdomains, contact sites, microscopy, mass spectrometry

## Abstract

Eukaryotic life is defined by the presence of organelles. Organelles, in turn, were classically defined as specialized membrane-bound compartments composed of a unique set of macromolecules which support specific functions. Over the last few decades, a concerted effort into uncovering which components are present in each organelle has shaped our view of cell biology. However, despite some organelles already being visualized over 100 years ago, we are still discovering new organelle residents. Furthermore, our concept of both ‘organelles’ and ‘compartmentalization’ has evolved together with our deepening understanding in a number of fields. These include: organelle substructure and organization; the network of contact sites which interconnects all organelles; and membraneless organelles and phase-separated condensates. This review explores how image- and mass spectrometry-based methods can be used to understand the spectrum of where components are localized: from complexes, to subdomains, and whole organelles. The components we mainly focus on are proteins of the mitochondria and secretory pathway organelles.

## 1 Introduction

**Figure 1:**
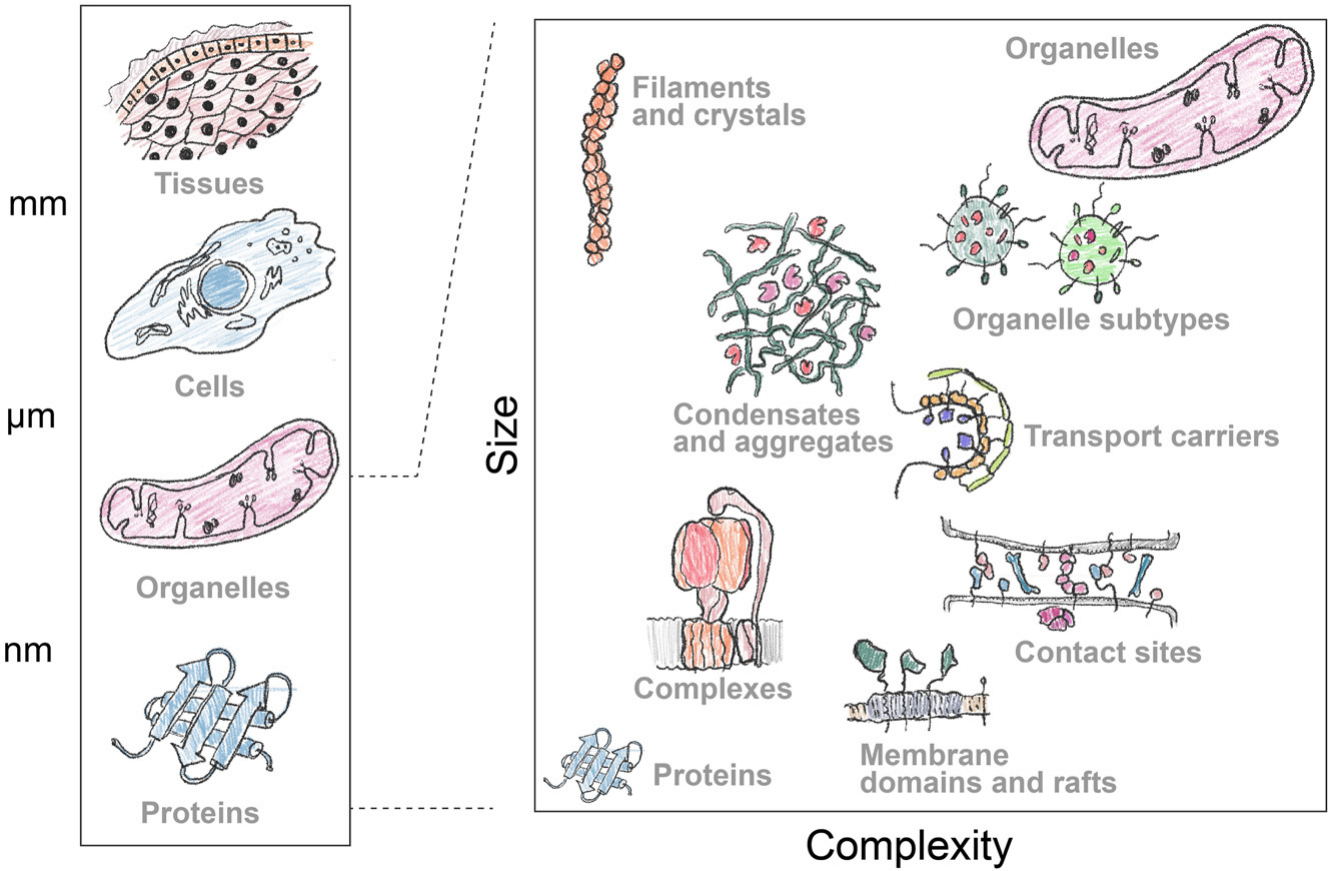
Apart from a few exceptions, the biological ‘units’ below the tissue level are not visible by eye. Microscopy paints a picture of how cells and organelles look, and new revolutionary tools such as AlphaFold, illustrate the other end of the size/complexity spectrum: what proteins look like. In between these two extremes, lies almost every possible combination of these two parameters. For some of these points robust methods exist which enable a clear picture of their composition to be built, such as identifying the components of protein complexes. However, other points along this continuum remain far more ambiguous, like organelle subdomains and subtypes, which at least in part are difficult to characterize due to the lack of a concrete definition. Furthermore, defining these subcellular regions is not trivial. For example, an organelle subdomain is composed of a unique collection of proteins (or protein complexes) and lipids to support a specific function (or set of functions). This may sound very much like the definition of an organelle. Their stability may also vary widely, and size would not be a helpful guide since an endoplasmic reticulum subdomain may be larger than some whole organelles. Instead of focusing on a definition, we suggest using and advancing the methodologies discussed below to provide localization and interaction context for subcellular regions and how they differ from their surroundings.

The textbook view of a eukaryotic cell was formed by seminal discoveries made in the middle of the previous century. The information flow goes from DNA to proteins (Crick 1958), which adopt defined structures to help them perform various cellular functions. Some cellular components are packaged into membranous compartments called organelles. Their shapes and interactions were observed by microscopy, and their contents were studied by biochemical fractionation (Claude 1946; de Duve et al. 1955; Palade and Porter 1954; Palade 1953; Porter et al. 1945). This resulted in a familiar ‘general map’ of the cell where organelles are filled with freely diffusing metabolites and protein complexes. The developments of new imaging and biochemical techniques led us to revise this perspective. There seems to be more organizational levels in between organelles and proteins that are not yet clearly defined (Figure 1).

The cytosol, the media between organelles, was previously viewed as an unstructured space. The notable recent discoveries that challenge this dogma include membraneless condensates. They can rapidly assemble to organize cytosolic components upon stress. Under normal conditions condensate formation is the main driving force behind the formation of nuclear subcompartments such as nucleoli (Lyon et al. 2021). The nucleus features many membraneless subcompartments but itself is a classic membrane-bound organelle. It is enclosed in the nuclear envelope which is a subdomain of the endoplasmic reticulum (ER).

The ER marks the beginning of the secretory pathway and is the largest and the most diverse organelle in many cells. It features multiple defined regions with distinct functions such as protein synthesis, transport, and lipid droplet and peroxisome biogenesis (Obara et al. 2023). What the mechanisms maintaining this compartmentalization are, and how many other ER functions are compartmentalized, remains poorly characterized. Peroxisomes and lipid droplets derive many of their proteins via the ER. These small organelles can also have specialized populations within one cell, or subtypes with specific functions (Eisenberg-Bord et al. 2017). Other secretory pathway organelles such as the Golgi apparatus, endosomes and lysosomes feature complex membrane shapes and dynamic behaviour. Membrane domains composed of distinct lipids and proteins that define this behaviour are very difficult to visualize because most microscopy methods lack the required spatial or temporal resolution. The ‘end’ of the secretory pathway connects to the plasma membrane. Unlike the depiction in schematic cartoons, it is not an unstructured sea of lipids and proteins as it also contains subdomains. These range from short-lived lipid rafts (Levental et al. 2020) to more stable and well-defined caveolae and eisosomes (Kefauver et al. 2024).

Endosymbiotic organelles such as mitochondria and chloroplasts are not connected to the trafficking events within the secretory pathway but both have complex ultrastructure. The multiple membranes of these organelles form subcompartments with distinct protein composition and functions (Morgenstern et al. 2017; Vögtle et al. 2017; Wietrzynski et al. 2020). The membranes that bear the electron transport chains maintain characteristic shapes. In mitochondria, the shape of the inner membrane is maintained by the mitochondrial cristae organizing complex (MICOS) (Pfanner et al. 2014). How the shape connects with functions such as metabolism and protein biogenesis remains to be fully investigated.

Finally, only recently has the importance of organelle connections been fully acknowledged. Most organelle pairs form membrane contacts that are mediated by specific mechanisms. They are essential to perform lipid and metabolite trafficking, and can affect the function and composition of the opposing organelle in the contact region (Scorrano et al. 2019; Valm et al. 2017).

Filling in the organizational levels between organelles and proteins is possible because of the development of mass spectrometry (MS)-based biochemical methods and imaging techniques (Breckels et al. 2024; McCafferty et al. 2024). In this review we highlight key experimental methods and their advances with one part devoted to biochemical approaches, and another to imaging by fluorescence microscopy (FM) and electron microscopy (EM). The different approaches discussed in the text are summarized in Table 1. We also outline the limitations and exciting new directions of method development. From a biological point of view, we largely focus on the application of proteomics and imaging to the studies of protein distribution, biogenesis and trafficking in the secretory pathway and mitochondria.

**Table 1:** Summary of the proteomics- and microscopy-based methods to study protein complex and organelle composition.

	Method	Key features	Advantages	Disadvantages	Application example	Further reading
	Proteomics					
	Immunoprecipitation (IP)	Proteins are recognized by antibodies which bind beads for precipitation	Most stably-interacting complex components can be identified	Weak or transient interactors are less likely to be captured	Sowa et al. (2009)	Huttlin et al. 2021; Michaelis et al. 2023
Proximity-labeling techniques	Pairwise biotin ligase and acceptor peptide: BirA/AviTag	A protein tagged with BirA can biotinylate a proximal protein tagged with AviTag	Transient interactions can be detected and readout is a streptavidin blot	No biotinylation if the lysine residue in AviTag is not accessible; challenging for lowly abundant proteins	Cohen et al. 2023; Mukhopadhyay et al. 2024	Beckett et al. 1999; Cronan 1990
	Promiscuous biotin ligases (BioID, TurboID, plus many others)	BioID or TurboID-tagged proteins biotinylate lysine residues in stable and transient proximal proteins	Multiple putative interactors can be identified from a single proteomics experiment	Lacks spatio-temporal control	Stockhammer et al. 2024b	Branon et al. (2018)
	Ascorbate peroxidase-based labelers (APEX and others)	APEX-tagged proteins biotinylate electron-rich side-chains in stable and transient proximal proteins	Fast, time-controlled biotinylation which can also be used to label RNA, and for EM imaging	Requires the oxidizing agent H_2_O_2_ which can be toxic	Sroka et al. (2025)	Martell et al. 2012; Rhee et al. 2013
	Other activatable proximity-labeling enzymes	Labeling of proximal proteins is induced by small molecules or light	Activators allow for time-controlled labeling with limited toxicity	Not all enzymes function equally well in each compartment; some background activity is present	Lee et al. (2023a)	Valor et al. (2025)
	Split proximity-labelers	Biotinylation of proximal proteins occurs when both fragments of the enzyme are complemented	Highlights the proximal interactome of proteins enriched at subdomains	Not all versions are reversible, thus not permitting dynamics to be monitored	Cho et al. 2020; Kwak et al. 2020	Martell et al. (2016)
Fractionation-based techniques	Size-exclusion chromatography fraction profiling	Solubilized complexes are separated according to their hydrodynamic radius	Hundreds of complexes can be analyzed at once	Prior knowledge of the complex(es) is required. Unstable and membrane complexes are not favourable	Bludau et al. (2023)	Heusel et al. (2019)
	BN-PAGE slice profiling	Intact complexes prepared in mild detergent are resolved by gel electrophoresis	Resolves native and active complexes, particularly good for membrane-embedded ones	Laborious protocols which require specialist equipment	Schulte et al. (2023)	Müller et al. (2016)
	Centrifugation-based fractionation	Subcellular compartments or complexes are separated based on their size, shape, and mass	Analysis of fractions can be coupled with correlative profiling to analyze multiple locations	Fractions are not ‘clean’ (e.g. organelles cannot be separated well)	Cox and Emili 2006; Foster et al. 2006	Christoforou et al. 2016; Itzhak et al. 2016
	Organelle-IP	A tagged membrane protein is used to isolate the target organelle from homogenized cells	Rapid protocol enables metabolome analysis; isolation is relatively clean	Protocols are laborious and require high precision	Hein et al. (2025)	Chen et al. (2016)

	Microscopy					

Fluorescence microscopy	Classic fluorescence microscopy	Fluorescently tagged protein is visualized in a widefield or confocal setup	Flexible tag choice; can be high-throughput	Resolution is limited by light diffraction	Litsios et al. 2024, Simpson et al. 2012	Bankhead 2025; Lee and Kitaoka 2018; Montero Llopis et al. 2021; Senft et al. 2023
	Targeted superresolution microscopy (SRM), e.g. STED	Excitation beam is spacially modulated to reduce excitation area	Can image living cells	High light intensities and limited fluorophore choice	Kondadi et al. 2020; Segawa et al. 2020	Jacquemet et al. 2020; Möckl and Moerner 2020
	Stochastic SRM (e.g. PALM, STORM, RESI)	Few target molecules with non-overlapping signals are imaged at the same time	High localization precision	More complex labeling and longer data acquisition	Reinhardt et al. 2023; Wang et al. 2019	
	MINFLUX	Patterned beam tracks individual fluorophores	High spatial and temporal resolution with low intensities	Complex instrumentation required	Deguchi et al. (2023)	
Electron microscopy	Electron tomography (ET)	Fixed and resin-embedded sample is imaged in the EM at different angles to reconstruct the 3D volume	High resolution view of organelle ultrastructure	Small field of view, no molecular specificity, fixed sample	Marsh et al. (2001)	McCafferty et al. 2024
	Slice-and-view EM	Thin slices of the sample are removed by FIB or knife and the surface is iteratively imaged by SEM	Large volumes can be imaged	Lower resolution than ET	Heinrich et al. (2021)	Xu et al. (2017)
	Correlative light and electron microscopy (CLEM)	Fluorescence and electron microscopy are applied to the same sample	Combines molecular specificity of fluorescence with a high-resolution view of the cell	Complicated protocols to re-locate the region of interest in electron microscopy	Kukulski et al. 2012a; Weigel et al. 2021	Scher and Avinoam (2021)
	*In-situ* cryogenic ET (cryo-ET)	Thin slices of vitrified cells produced by knife or FIB are imaged with ET under cryogenic conditions	Shows the view of the organelles, structures and positions of macromolecules in their near-native state.	Expensive and laborious; only large molecular complexes can be identified	Mühleip et al. 2021; Waltz et al. 2025	Berger et al. 2023; McCafferty et al. 2024

## 2 Mass spectrometry-based biochemistry: from molecules to organelles

**Figure 2:**
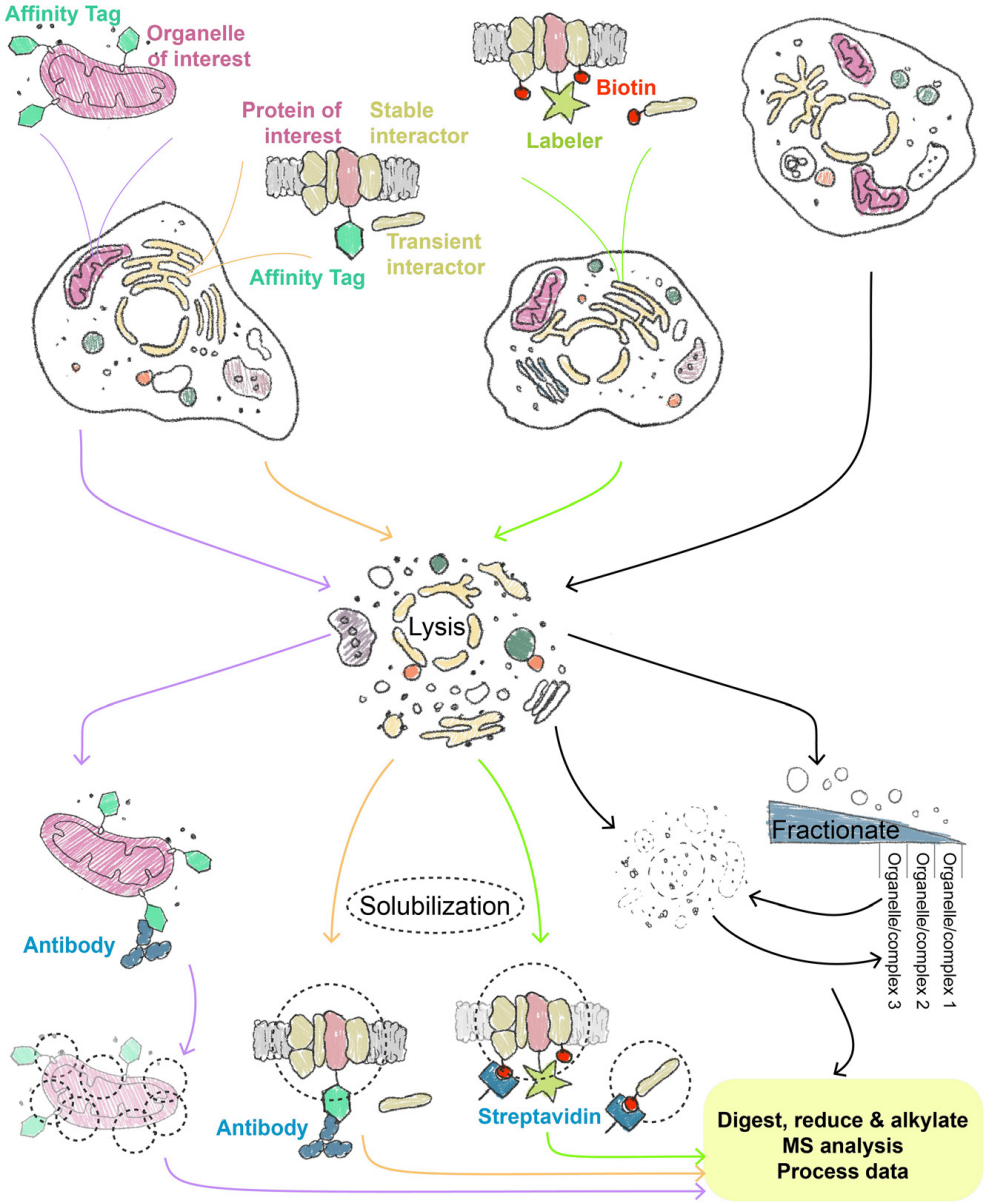
The mass spectrometry (MS)-based biochemical methods to study protein interactions and localizations across scales. To discover proteomes (or lipidomes and metabolomes) of organelles, membrane proteins with a taggable terminus facing the cytosol can be fused to affinity tags and used for enrichment (Section 2.4). In these Organelle-immunoprecipitation (IP) workflows (shown by the purple arrows), cells are lysed gently to break the plasma membrane but preserve organelle structure, and the organelles are immunoprecipitated by bead-bound antibodies. The captured organelles are then solubilized and processed for MS analysis. Membrane-bound or soluble proteins of interest (POIs) can be tagged in essentially the same way, but if the cells are simultaneously lysed and solubilized (often in mild detergents to help maintain protein interactions), then bead-antibody conjugates can be used to pulldown the bait and its associated proteins (Section 2.1, orange arrows). A similar process can be followed to find transient or weak interactors and spatial information, by tagging POIs with proximity labeling (PL) tools (Section 2.2, green arrows). Here lysis and solubilization conditions are generally quite harsh since interactors that came into proximity of the tagged POI will be biotinylated and therefore detected by streptavidin even when protein interactions are dissociated. Lastly, cells which do not express any tagged protein can be used to map protein localization in the context of either complexes or organelles, via fractionation-based techniques (Section 2.3, black arrows). Depending on whether the cells are gently lysed or solubilized in mild detergent, organelles or complexes can be resolved, respectively. Fractionated organelles need solubilizing prior to MS analysis, just as with Organelle-IPs.

If cellular substructure and organization were perfectly resolved, locating any protein in a cell map would be easy. For each protein, one would be able to quickly find out its working environment: which co-workers (co-factors) it interacts with for longer (stable) or shorter (transient) periods of time; which room (subdomain) it functions in; and in which factory (organelle) this room is. Thanks to the developments in MS-based biochemical methods over the last decades we have the means to discover all of the above spatial information for an increasing number of proteins in a wide range of model organisms. Here we will explore these techniques and their applications, ranging from: performing pulldowns to find cofactors; using proximity labeling tools to uncover transient interactors and explore subdomains; and leveraging ‘classic’ and cutting-edge approaches to probe whole organelles (Figure 2).

### 2.1 Discovering stable protein interactors and components of complexes

Finding non-covalently bound cofactors and stable interactors of a protein of interest (POI) describes what a protein complex looks like, and from this one can make strong functional hypotheses and inferences. The process of finding a protein’s cofactors, which may assemble into a multi-subunit complex, often consists of the following steps: (1) cell lysis and membrane solubilization with a solution of detergents, salts and protease inhibitors, optimized to preserve protein-protein interactions (PPIs); (2) enrichment of a POI and associated interactors with antibodies bound to beads; and (3) digestion of this enriched ‘fraction’ by peptidases to yield peptides whose identity can be revealed by MS analysis. Step 2 (enrichment) can be achieved using antibodies raised against the native POI, however, it is more often performed with antibodies against a tag fused to the POI (or POIs). Short tags, such as HA, FLAG^®^, Myc or S-tag – none of which are more than 15 amino acids – minimize interference with the POI’s function. Other tags, such as green fluorescent protein (GFP) or SNAP-tag^®^, are much larger, however, can also be visualized by microscopy and thus serve a dual-purpose. The antibodies against these tags are often purchased pre-conjugated onto beads, whereas antibodies against endogenous proteins can be coupled to beads during the enrichment protocol. Either way, the beads allow for ‘pulldown’ or *immunoprecipitation* (IP), that separates the POI and its interactors from the rest of lysate.

Every field of cell biology has benefitted from variations of the above pipeline, and such methods have contributed to countless breakthrough discoveries. One such field is that of ubiquitin and its dependent processes and pathways. In 2009, high-content IP-MS of 75 virally-expressed, FLAG-HA-tagged deubiquitinating enzymes was used to reveal 774 candidate protein cofactors (Sowa et al. 2009). Subsequent studies utilized similar approaches to characterize the stable protein interaction landscapes formed by: the Cullin-RING ubiquitin ligases (Bennett et al. 2010); membrane-associated players in ER-associated degradation (Christianson et al. 2012); adaptor proteins containing ubiquitin X domains (Raman et al. 2015); and the family of over 20 E3 ubiquitin ligases embedded in the ER membrane (Fenech et al. 2020).

All of the above works included the parallel sample processing of multiple baits (the terms used to describe the tagged POI recognized by the antibody) and this set-up is essentially internally-controlled when it comes to the analysis step. The analysis pipelines developed and utilized in these studies are Comparative Proteomic Analysis Software Suite (CompPASS) (Behrends et al. 2010; Sowa et al. 2009) and the affinity-enrichment (AE)-MS method (Keilhauer et al. 2015). Both pipelines rely on the presence of a relatively consistent background ‘proteome’ across multiple samples (such as proteins non-specifically captured in the bead matrix), which allows for enrichment scores and interaction likeliness to be calculated with increased confidence.

It is notable that both CompPASS and the AE-MS strategies form the analytical foundation for landmark, high-content interaction datasets. The former was employed for generating the BioPlex Network. This project began in 2015 (Huttlin et al. 2015) and currently describes the interaction partners for around half of the proteome in cultured human cells (Huttlin et al. 2021). AE-MS, on the other hand, was employed for the global interactome analysis of baker’s yeast (Michaelis et al. 2023). Here, the authors performed successful pulldowns of approximately two-thirds of all expressed yeast proteins, which were endogenously tagged at their C-termini with GFP. The strains expressing the tagged proteins were from the original whole-genome GFP collection (Huh et al. 2003). The enormity of this study, which discovered over 30,000 interactions, revealed the interconnectivity between protein complexes, and such data can be used to extract meaningful information about how multi-subunit machines assemble into organellar subdomains.

These kinds of analysis are becoming more common and accessible through the availability of highly advanced instruments, capable of deep-proteome mining over very short gradients. For example, the Orbitrap-Astral MS can assay 200 samples per day, with each sample being analyzed over a seven-minute gradient (Serrano et al. 2025). Therefore, while probing stable interactions between components of protein machines has been accessible for several decades, the multi-dimensional advances of these proteomic methods mean that their ‘standard’ output will transcend the realm of complexes into that of higher-order suborganellar structures.

### 2.2 Using proximity-labeling methods to go beyond complex components

PPIs that are too weak or transient will not be captured by the methods above. Therefore to detect weakly-bound components of molecular complexes, enzyme substrates, or clients of protein transport and quality control pathways, other methods are needed. These include stabilizing such interactions by constructing ‘trapping mutants’ of an enzyme, or by using chemical cross-linking. While trapping mutants are limited by the fact that they have to be made on an individual basis, cross-linking is more popular (Hevler and Heck 2023). Another approach however, is proximity labeling (PL), where spatial proteomes and transient or weak interactors are marked *in vivo* and purified later. Due to its simplicity and universality, PL is a widespread technique and will be the focus of this section. PL for protein landscape mapping generally concerns tagging a POI with a ‘proximity labeler’. These labelers activate compounds that covalently bind to (or label) specific amino acid residues within ∼10–30 nm, and this, in turn, enables their capture and identification. Usually, the label is a form of biotin and capture is based on the high-affinity biotin-streptavidin interaction. There are two main classes of labelers: those based on biotin ligases, and those based on peroxidases. The latter are discussed in Section 2.5.

Long before the terminology ‘proximity labeling’ was coined, the bacterial biotin ligase BirA was known to uniquely biotinylate one specific lysine within a short peptide sequence (Avi, or AviTag) in an ATP-dependent manner (Beckett et al. 1999; Cronan 1990). With biotinylated AviTag being efficiently captured by streptavidin, the BirA-Avi approach is thus very powerful for discovering, detecting and validating pairwise protein interactions – including transient enzyme-substrate interactors which are not stably associated with the POI. To broaden the scope of biotin ligases beyond pairwise assays, Roux and colleagues developed BioID (Roux et al. 2012). This method leveraged the reactivity of a promiscuous version of BirA (R118G) (Choi-Rhee et al. 2004), which was fused to Lamin-A and thus used to label proximal nuclear envelope proteins in human cells (Roux et al. 2012). This BirA mutant works by activating biotin in the presence of ATP, generating a cloud of reactive biotinoyl-5′-AMP species which can covalently bind to the amino group in lysine residues (Choi-Rhee et al. 2004). Upon cell solubilization, biotin-labeled proteins can then be captured and enriched by streptavidin-coated beads, and after digestion, their identity can be revealed by MS analysis. BioID has been fused to a number of different baits in different model systems (Qin et al. 2021) and used in high-content protein landscape screening. The latter approach was used to shed light onto the relationship between the centrosome and the cilium (Gupta et al. 2015), and proteins associated with mRNA-related processing in stress granules and processing bodies (Youn et al. 2018). In a seminal study, BioID mapped the proximal interactome of nearly 200 proteins in human cells (Go et al. 2021). This dataset can be explored online (cell-map.org) and was used to uncover proteins resident at the ER-mitochondria contact.

Despite these successes, the activity of BioID and the smaller BioID2 (Kim et al. 2016) is limited, especially at lower temperatures. Thus, in model organisms like baker’s yeast, which is grown at 30 °C, the application of BioID was restricted to a handful of studies such as those interrogating the interactions of proteins associated with the ribosome (Opitz et al. 2017) and mitoribosome (Singh et al. 2020). However, in 2018, a much more active promiscuous biotin ligase, called TurboID, was developed in the Ting laboratory (Branon et al. 2018). This ligase was engineered by direct evolution of BirA in yeast, and thus displayed higher activity at a much wider range of temperatures. TurboID has been used in: fission yeast to reveal interactors of soluble ribosomal and RNA exosome proteins (Larochelle et al. 2019); baker’s yeast, to study vesicular transport (Schoppe et al. 2020; Vargas Duarte et al. 2022); mice, to label the secretome by fusion to a SEC61 translocon component (Kim et al. 2021); and in human cells, to explore points of contacts made by the ER with either endosomes (Wu and Voeltz 2021) or mitochondria (Nguyen and Voeltz 2022). These represent a small fraction of TurboID-related works since this approach has made a far-reaching impact in a wide range of biological fields.

In addition to BioID and TurboID, there are a variety of different promiscuous biotin ligases to choose from, including, but not limited to: BASU (Ramanathan et al. 2018); miniTurboID (Branon et al. 2018), microID2 (Johnson et al. 2022) and ultraID (Kubitz et al. 2022) – all of which are smaller than TurboID; and AirID (Kido et al. 2020). Most studies in animal cells have used these promiscuous biotin ligases fused to an exogenously expressed POI, however more recent works have moved towards CRISPR/Cas9-based endogenous tagging, to mitigate artefacts and limitations associated with protein overexpression. A recent example includes the development and optimization of knock-in methods for native C- and N-terminal TurboID tagging (with both options preserving endogenous promoters). By tagging the *μ* subunits of four adaptor protein (AP) complexes involved in membrane trafficking, shared and unique putative interactors and cargo for each of the complexes were identified (Stockhammer et al. 2024b). This information will help shed light onto the distinct localization of the AP complexes to specific compartments of the endo-lysosomal system and the subtly different roles they play in cargo sorting. In another example, spatially-distinct proteins of the ER were endogenously tagged with TurboID (Merta et al. 2025). This work could distinguish unique suborganellar proteomes of the ER and focussed on the differences between its perinuclear sheets and the tubular network. Furthermore, the protein calmin was identified as an ER tubule-specific protein required for actin tethering.

While endogenous tagging methods are becoming more accessible, it is still challenging and time-consuming to generate several knock-in human cell lines. This is not, however, the case in baker’s yeast, due to the ground-breaking construction of SWAp-Tag (SWAT) whole-genome collections of strains (also called libraries). These collections enable the generation of *new* ones, in which virtually every gene in the genome is endogenously fused to a desired tag of choice (see Section 3.1) (Weill et al. 2018; Meurer et al. 2018; Yofe et al. 2016). Using this method, five proteome-wide collections were made for BirA, AviTag, BioID and TurboID, with an additional TurboID library generated containing a system to enhance biotin-specific signal-to-noise. This system works by inducibly reducing the high background biotinylation levels which exist in yeast (Fenech et al. 2023). Strains from this whole-genome library were used to uncover functions and regulatory components for a family of ER-resident contact site proteins, by profiling their proximal protein environment (Fenech et al. 2025). Furthermore, the TurboID construct in these libraries also contains an HA-tag which allows for both anti-HA and streptavidin-based pulldowns to be carried out from the same strain, similar to an approach earlier proposed with BioID in mammalian cells (Liu et al. 2018). By using this combined approach on a TurboID-HA-tagged component of the EMC (ER-membrane complex) insertase, it could be deduced which putative interactions found by TurboID were stable, since they also appeared in the ‘classic’ HA pulldowns. By extension, proteins *only* found by TurboID, were deemed more likely to be transiently-interacting substrates of the EMC, and indeed this was validated using the pairwise BirA-AviTag approach (Fenech et al. 2023).

While the power of the BirA-AviTag pair for protein interaction validation is clear, it can also be used for high-content interaction screening. This was done in yeast, using strains from the BirA library (Fenech et al. 2023) expressing either one of two tagged ER translocation channels (Sec61 or Ssh1). These strains were then crossed to the whole-genome AviTag library, and so any AviTagged protein which came sufficiently close to either Sec61 and/or Ssh1 would be labeled with biotin. The lysate from these strains was subsequently used for high-throughput, streptavidin-based dot-blotting to determine relative, proteome-wide protein interaction ‘strength’ for both Sec61 and Ssh1 (Cohen et al. 2023). This identified the types of secretory pathway clients which preferred to transiently interact with Sec61 or Ssh1 to translocate into the ER.

Aside from yeast, BirA-AviTag has also recently been leveraged for client discovery of human E3 ubiquitin ligases (Mukhopadhyay et al. 2024). In this method, BirA fused to an E3 ubiquitin ligase is expressed from a plasmid, which together with another plasmid encoding ubiquitin-AviTag, are simultaneously transfected into cells. This way, the tagged E3 of interest can biotinylate the AviTag module upon transfer of ubiquitin to its substrates, thereby enabling their capture and identification. This exciting approach was successfully implemented for three distinct, soluble E3 ligases.

One limitation with all biotin ligase-based PL tools is that biotin (which is vitamin H) is an essential component of all media used to grow cells and therefore labeling starts as soon as TurboID is translated and folded. This lack of spatio-temporal control can result in decreased labeling specificity, however, several methods have been developed to overcome this limitation. The first approach is based on the class of biotin ligases which are ‘split’ into two fragments, ensuring that only upon fragment complementation can biotinylation occur. Split-BioID and split-TurboID have both been used to investigate the ER-mitochondria contact site by fusing each ‘half’ of the split-biotin ligase to proteins embedded in either membrane (Cho et al. 2020; Kwak et al. 2020) and revealed novel players at this contact. The second approach is to create ‘activatable’ biotin ligases. For example, photoTurbo, LOV-TurboID and chemoTurbo respectively rely on UV light, blue light, or small molecules for rapid and time-specific activation (Lee et al. 2023a; Liu et al. 2021). Several other forms of photo-activatable PL enzymes exist (Valor et al. 2025). Furthermore, methods development in enhancing spatio-temporal resolution includes tools that are reactive and location-specific which do not rely on protein tagging (Geri et al. 2020; Shiraiwa et al. 2020). In general, the versatility and adaptability of PL makes these approaches a very powerful toolset for interrogating spatial information and interactions that can otherwise be challenging to capture.

### 2.3 Fractionating cells into subcellular structures across scales

Fractionation-based techniques can be used to collect information about protein localization across multiple scales: from protein complexes to whole-organelles; dual-locality; and the relocalization of proteins upon changes in conditions. The concept of separating ‘fractions’ is founded on the differentiation of cellular ‘objects’ based on their biophysical and biochemical properties. The type of fractions that are collected depend on how the cells are lysed prior to separation, and the method of fractionation used.

To analyze medium to large protein complexes, cells can be lysed and solubilized in mild detergent to preserve stable PPIs, and fractionated by size-exclusion chromatography, which separates components based on their hydrodynamic size. Heusel, Bludau and colleagues collected 81 fractions, which following tryptic digest were analyzed by a custom MS and data analysis pipeline *CCprofiler* (Heusel et al. 2019). This allows proteins with similar elution profiles to be matched and correlated to a complex, and thus a picture of nearly 500 protein assemblies in human tissue culture cells was built. A detailed protocol is also available for this methodology (Bludau et al. 2020) and a similar approach can be used to identify changes in complex components (Bludau et al. 2023). Another way that works particularly well in separating medium to large protein complexes embedded into membranes is Blue-native polyacrylamide gel electrophoresis (BN-PAGE) (Müller et al. 2016). In a recent study purified mitochondria were solubilized with the mild detergent digitonin and separated on BN-PAGE (Schulte et al. 2023). The resulting gel was cut into thin slices and their contents analyzed by MS. The resulting individual protein profiles were correlated to identify molecular complexes. The study led to the discovery of new quality control factors at the mitochondrial outer membrane translocase.

On the other hand, cells can be lysed but with membranes and organelles kept (semi-) intact. This non-solubilized lysate can be separated by different types of fractionation method, and afterwards, be solubilized to extract proteins for MS analysis. One of the simplest types of fractionation is differential centrifugation. This relies on a series of centrifugation steps that separate cellular components based on their sedimentation speed that depends on mass, size, and shape. Mechanically homogenized mouse tissue subject to this separation strategy results in the collection of fractions representing the nucleus, cytosol, mitochondria, and organelles of the secretory pathway (Cox and Emili 2006). Other protocols which are designed to collect multiple organelle fractions include sonication-based lysis followed by discontinuous sucrose gradients to isolate ER, mitochondria, and mitochondria-associated membranes, another ER subdomain (Williamson et al. 2015). A fine sampling and proteomic analysis of continuous sucrose gradient fractions was used to characterize protein relocalization in non-alcoholic fatty liver disease hepatocytes (Krahmer et al. 2018). Some protocols also combine methods – for example, a differential centrifugation fraction can be subject to further enrichment by using an antibody against a protein resident in the organelle of interest. This approach has been used for mitochondria and stress granule isolation (Afanasyeva et al. 2018; Wheeler et al. 2017). Differential and density-based centrifugation can be integrated, for instance, to isolate Golgi stacks (Graham 2001).

A limitation of this methodology is that due to extensive membrane contact site formation in cells, the above fractions do not represent a ‘clean’ isolation and include varying degrees of ‘contaminants’ from other subcellular locations. However, instead of collecting specific fractions in which specific organelles are enriched, an alternative is to simply collect many fractions from cell lysate and analyze them all. In a landmark study, homogenized mouse liver was fractionated by sucrose gradient and 32 fractions were collected and processed (Foster et al. 2006). Known organelle markers produced a characteristic MS intensity profile across these fractions, and these in turn were used to benchmark all other detected proteins to identify their localization(s). This principle of protein correlation profiling (Andersen et al. 2003) is similar to the protein complex profiling by size exclusion chromatography described above (Heusel et al. 2019), with one main difference coming down to whether fractionation happens before or after solubilization, respectively.

Protein correlation profiling is a very powerful strategy and has been applied, for example, to define the proteome of lipid droplets (Krahmer et al. 2013) which are challenging organelles to isolate. The concept of correlative profiling forms the foundation of several other methodologies including LOPIT (Localization of Organelle Proteins by Isotope Tagging) and its derivatives, and dynamic organelle mapping (Itzhak et al. 2016). LOPIT combines fractionation with isotope labeling (which enables accurate quantitation) and it was first applied to uncover proteins localized to the ER and Golgi in plants (Dunkley et al. 2004). Since it was first published, several advances to the LOPIT pipeline have been made, such as hyperLOPIT, which relies on extensive fractionation and advanced, multiplexable isobaric labelers to increase the resolution of this spatial proteomics method (Christoforou et al. 2016; Mulvey et al. 2017). Thus, while fractionation-based techniques may be more challenging to adapt to high-throughput methods, their utility in assaying a wide spectrum of subcellular locales, makes them an indispensable part of the protein localization toolkit.

### 2.4 Enriching whole organelles with Organelle-IP strategies

Organelle-IPs describe a set of protocols in which cells are collected and lysed in a ‘gentle’ potassium-based buffer that does not solubilize the membranes. This preparation method and the lack of detergents ensures that organelles can be isolated from this homogenate by IP of an epitope-tagged protein embedded in the membrane of the organelle of interest. The isolated structures can then be processed for differential MS-based analyses to characterize their proteomes, lipidomes or metabolomes – or any combination of these. One of the most advantageous features of these Organelle-IPs is that they are rapid: going from homogenate to extraction of the macromolecules of interest takes around 10 minutes, ensuring as close a proxy to the native composition in living cells as possible. This is a particularly important consideration for metabolites, which can ‘leach’ out of organelles even in samples kept at low temperature. This in turn renders fractionation-based methods (see Section 2.3) less amenable to this type of organelle profiling.

The first Organelle-IP protocol to be established concerned mitochondria, where an outer mitochondrial protein, OMP25, was ectopically expressed in HeLa cells and fused to a 3 × HA-GFP construct to allow for both IP and visualization, respectively (Chen et al. 2016). The authors confirmed that the isolated mitochondria were intact and had a functional membrane potential. Immunoblot analysis showed that the ‘Mito-IP’ sample gave a highly specific mitochondrial profile relative to a sample prepared by differential centrifugation, highlighting the potential level of purity of this type of organellar isolation. There does, however, appear to be some contamination from peroxisomes in the step-by-step guide to performing Mito-IP experiments (Chen et al. 2017). Purified mitochondria subject to metabolomic analysis found metabolites previously associated with the mitochondria, as well a novel metabolite, ADP-ribose (Chen et al. 2016). Further metabolic profiling following treatment of the cells with different respiratory chain inhibitors (used to approximate various disease-states) also revealed local changes to the mitochondria, which would have been masked by whole-cell profiling. This included dramatic compartment-specific increases in ratios of NADH to NAD^+^ and oxidized to reduced glutathione, which both play a crucial role in maintaining redox homeostasis.

Following hot on the heels of the Mito-IP protocol, was the Lyso-IP method for the enrichment and purification of lysosomes from human tissue culture cells (Abu-Remaileh et al. 2017). Here, the lysosomal membrane protein TMEM192 was tagged with 3 × HA and resulting IPs displayed high specificity for known lysosomal proteins, with very little signal from proteins of other organelles. Metabolomics of lysosomes purified from cells in which either the V-ATPase or mTOR kinase activity was inhibited, uncovered a role for lysosomes as a storage compartment for either non-essential or essential amino acids, respectively.

Establishing fast protocols for organelle capture of high purity gave way to the expansion of downstream applications in addition to metabolic profiling. In 2019, transgenic mice expressing MITO-Tag (the same as the original Mito-IP construct (Chen et al. 2017, 2016)) were generated and Mito-IP samples from liver tissue were used for multi-omic analyses to assay their proteome, lipidome and metabolome (Bayraktar et al. 2019). Here, the authors found that in mice that underwent fasting, alanine was sequestered in hepatocyte mitochondria, despite a decrease in total levels from liver tissue. Other recently-established Organelle-IP protocols include: PEROXO-IP, where a segment of Pex26 was tagged with 3 × HA-GFP (Ray et al. 2020); Endo-IP, where early endosomal marker EEA1 was FLAG-tagged (Park et al. 2022); and Golgi-IP, where the Golgi-resident TMEM115 was fused with 3 × HA (Fasimoye et al. 2023). Notably, proteomic analysis of Endo-IP samples identified putative early endosomal cargo proteins (Park et al. 2022). Furthermore, by combining Endo-IP with Lyso-IP and a proteomics workflow optimized for detecting cleavage fragments of amyloid precursor protein (APP), differentially processed APP forms could be traced across different endolysosomal compartments (Park et al. 2022). Similarly, proteomic profiling of Golgi-IP samples found several, putative, novel Golgi-resident proteins, one of which, CCDC126, was confirmed to co-localize with Golgi marker proteins (Fasimoye et al. 2023).

Using endogenous antibodies, or combining CRISPR/Cas9-based engineering to generate cell lines where the protein used to capture an organelle is endogenously tagged, can help eliminate artefacts associated with exogenous expression. The latter approach was successfully executed in human stem cells, where natively tagged EEA1 and TMEM192 were used for combined Endo-IP and Lyso-IP to profile the endolysosomal system upon differentiation of these cells into neurons (Hundley et al. 2024). In a recent landmark study, CRISPR/Cas9 was used to fuse a 3 × HA tag onto 37 proteins resident across 19 different subcellular localizations (Hein et al. 2025). The localizations included: each organellar membrane; the plasma membrane; condensates; the centrosome; and five different assemblies (such as the proteasome and ribosome). Each of the generated cell lines was subject to Organelle-IP sample preparation to generate a subcellular map of over 7,600 proteins. This map details interactions between cellular compartments and was used to assign the localization of tens of proteins that were previously uncharacterized. Lastly, endogenous antibodies were employed in the ‘tagless’ Lyso-IP method, where an anti-TMEM192 antibody was used to enrich and metabolically analyze lysosomes from blood cells isolated from patients with a lysosomal storage disorder (Saarela et al. 2024).

Since organelle contact sites are retained after cell lysis, Organelle-IP has constraints similar to regular fractionation techniques. For example, despite the ER being included in the multi-Organelle-IP study (Hein et al. 2025), proteins from the mitochondria and peroxisome were highly enriched to levels close to, or even exceeding, ER-resident protein enrichment. This is likely due to factors that make an ER-IP particularly challenging, such as the size of this organelle and its high degree of interconnectivity with other organelles through numerous membrane contact sites (Valm et al. 2017). However, using one specific protein as a ‘purification handle’, makes Organelle-IPs distinct from the separation approaches based on physical and chemical characteristics of compartments. It is possible that such specificity enriches not the entire population of the organelle of interest, but perhaps a specific population (or subtype), or even a subcompartment. Taking the Golgi-IP as an example (Fasimoye et al. 2023), immunoblot analysis comparing the controlTAG and GolgiTAG cells showed a much stronger enrichment of GM130, a *cis-*Golgi marker, relative to GOLGIN97, a *trans*-Golgi marker. All together, this relatively new generation of subcellular profiling protocols represents an exciting opportunity to link multiple ‘-omics’ analyses to gain a new level of understanding in molecular organization.

### 2.5 Bridging biochemical and image-based methods with peroxidase-based proximity-labeling

Earlier, we reviewed promiscuous biotin ligases and how these PL enzymes have been extensively used to characterize a wide range of protein landscapes from organelles to protein complexes and unique transient interactions. However, the other half of the PL family concerns peroxidases – with APEX being one of the most widely-used tools. Engineered ascorbate peroxidase (APEX) was developed in 2012 as a protein tag to enable EM of subcellular regions, such as the mitochondrial matrix (Martell et al. 2012). Its peroxidase activity leads to the conversion of a substrate compound into electron-dense precipitate that can be visualized by EM. However, APEX can work with multiple substrates, and when used together with biotin-phenol and H_2_O_2_, functions as a PL enzyme which catalyses the conjugation of biotin onto proximal electron-rich amino acids such as tyrosine and tryptophan. Similar to BioID/TurboID-based proteomics, the proteins are enriched on streptavidin-conjugated beads after cell lysis. This approach was first used to map nearly 500 proteins to the matrix and the inner membrane of the mitochondria (Rhee et al. 2013).

A more active version of APEX, APEX2 (evolved by yeast surface display), was able to effectively biotinylate at lower expression levels (Lam et al. 2015). It has proved to be a powerful tool for both EM- and proteomics-based experiments of inner mitochondrial subcompartments (Hung et al. 2014; Lam et al. 2015) and for the cytosolic-facing domains of both the ER and mitochondria and the contact between them (Hung et al. 2017). APEX2 has also been used to characterize the proteomes of lipid droplets (Bersuker et al. 2018), and stress granules (Markmiller et al. 2018). Furthermore, other advances facilitate the use of this PL strategy to a broader range of questions. For example, a mutation in APEX2 was designed to disrupt a nuclear export signal in its sequence, while preserving its peroxidase activity, thus enabling this method for more effective nuclear-based labeling (Becker et al. 2023). A very recent development is that of iAPEX, which merges APEX2 with a D-amino acid oxidase capable of producing a local pool of H_2_O_2_ to minimize any toxicity associated with adding this strong oxidizing agent (Sroka et al. 2025).

Just like there are split versions of BioID and TurboID, split-peroxidase systems have also been developed. The engineered split-HRP can be used to label proximal proteins, and for parallel visualization purposes (Martell et al. 2016). Complementation of each ‘half’ of the split module localized to either side of neuronal synaptic clefts was observed by both FM and EM (Martell et al. 2016). Split-APEX, on the other hand, has been utilized to analyze ER-mitochondria contact sites (Han et al. 2019) and homo-dimerization of proteins at the ER and plasma membrane (Xue et al. 2017).

While the localization of proteins tagged with promiscuous biotin ligases is often imaged using conventional microscopy (courtesy of a short, tandem tag such as V5) (Branon et al. 2018), TurboID can be combined with fluorophores to enable more advanced visualization methods. For example, a ‘double-split’ system designed in yeast, consisting of both split-TurboID and split-GFP, was used to examine ER-mitochondria contacts with FM and proteomics (Fujimoto et al. 2023). Additionally, TurboID fused to a GFP-binding nanobody was directed to SARS-CoV-2 viral proteins tagged with GFP and which are localized to ER-derived structures in human cells (Lee et al. 2023b). Lastly, a light-inducible split-BirA/AviTag approach (called ALIBi for AviTag-specific Location-restricted Illumination-Enhanced Biotinylation) was developed and used to uncover distinct ribosomal subpopulations at mitochondrial and ER surfaces (Zhang et al. 2025). Proteomics identified a specific factor, Lsg1, required to recruit 60 S ribosomes to the latter membrane. Further development of dual-purpose PL tools is an exciting avenue to bring together the worlds of proteomics and imaging.

## 3 Microscopy: from organelles to molecules

The dream of a cell biologist is to see every molecule in the natural dynamic environment of living cells. The Ideal Microscope would highlight proteins that comprise organelles and their subdomains and how they got to their destinations. A wide range of microscopy techniques offer benefits and compromises in terms of resolution, speed and specificity. Here we go from low resolution methods that allow visualization of whole organelles to high-resolution techniques that can show individual molecules inside native cellular environment. We consider traditional low resolution FM that offers the highest throughput, the challenges of super resolution microscopy (SRM), and new promises of cryogenic EM for the investigation of organelle subdomains.

### 3.1 Proteome exploration by high-throughput microscopy

FM is a very sensitive technique because it detects only the light emitted by a fluorescent molecule specifically attached to the molecule of interest. Only a few emitted photons may be enough for visualization. The two main approaches for visualizing a cellular structure are: expressing a genetically encoded label, or using a dye-conjugated antibody applied to a permeabilized and fixed cell. Conventional widefield and confocal microscopes that are universally available usually do not require a particular type of fluorescent label and thus the labeling strategy is defined by the biological question. The label type becomes more important for SRM and is discussed in the next Section 3.2.

FM data are ubiquitous in organelle biology studies (Bankhead 2025; Lee and Kitaoka 2018; Montero Llopis et al. 2021; Senft et al. 2023). Punctate structures such as organelle contact sites or vesicles can be readily visualized under a widefield microscope and as little as 20 GFP molecules can be quantified and their position precisely determined (Picco et al. 2015; Wozny et al. 2023). Another way to get more insights from traditional FM is to leverage its throughput. Imaging on a widefield or a spinning disc confocal microscope is particularly fast and thousands of samples can be imaged automatically in multi-well plates. This approach can be combined with the power of yeast genetics to make systematic collections (libraries) of knock-in and loss-of-function mutants covering the whole genome. For example, tagging every protein with GFP yielded one of the first comprehensive cellular protein localization maps (Huh et al. 2003). Many proteins showed ‘punctate’ patterns indicating potential new localizations or organelle domains. More detailed studies of the GFP collection revealed proteome-wide localization dynamics under cell cycle progression and stress (Breker et al. 2013; Chong et al. 2015; Litsios et al. 2024). Using the synthetic genetic array procedure (Cohen and Schuldiner 2011; Yan Tong and Boone 2006), such collections can be modified by introducing an organelle marker of different color and additional mutations to all the strains to investigate the composition and assembly mechanisms of the known subdomains. For example, this was used to discover that the poorly characterized proteins Ldo16 and Ldo45 maintain the formation of a specific population of lipid droplets localized close to nuclear-vacuolar junctions (Eisenberg-Bord et al. 2017). A similar approach revealed that the nucleus-mitochondria contact mediated by a new ER protein, Cnm1, is maintained by the translocase of the outer mitochondrial membrane receptor Tom70 (Eisenberg-Bord et al. 2021).

While mutations or reporters can be simultaneously introduced into all the strains of the GFP collection (Huh et al. 2003), the tag itself cannot be switched to another. This changed upon the introduction of a new generation of collections called the SWAp-Tag (SWAT) libraries (Meurer et al. 2018; Weill et al. 2018; Yofe et al. 2016). They have every gene endogeneously tagged with a specially-designed acceptor cassette that can be exchanged to any other tag of choice within a time-frame of only two months. SWAT collections exist as two versions: a C-terminal one (C-SWAT) where native promoters are maintained, and an N-terminal one (N-SWAT) where promoters can be substituted. The latter collection allows for the creation of strains where each protein is both over-expressed and tagged with a fluorescent protein. This widens the possibilities of traditional yeast screens that use knock-out mutants to look for the loss-of-function since overexpression can find new phenotypes through gain-of-function. This approach was used to find new components and regulators of contact sites. Six different split-fluorescent protein contact site reporters were introduced into the N-SWAT collection where every protein was tagged with mCherry and overexpressed (Castro et al. 2022). Some of the reporters represented relatively poorly studied contacts made by peroxisomes with the Golgi apparatus and vacuoles. The study revealed 158 potential new residents and regulators of contact sites including Hob1, Hob2, and Csf1 that belong to the Vps13/ATG2 superfamily. This recently characterized superfamily comprises proteins that form large tubules that might shuttle lipids between membranes. The ability to quickly change tags in whole-genomic collections opens a broad range of opportunities in applying new imaging techniques to illuminate different aspects of organelle biology.

Loss-of-function screens on the background of a fluorescent reporter can be performed in mammalian cells. For example, the function and morphology of the secretory pathway organelles was studied with small-interfering RNAs covering the whole genome. The work identified 554 potential regulators of secretion and Golgi apparatus distribution (Simpson et al. 2012). More efficient and cost-effective pooled screens using gene silencing with CRISPR/Cas9 were used together with ratiometric reporters measured by flow-cytometry to identify MTCH2 as a protein insertase in the outer mitochondrial membrane (Guna et al. 2022). The full power of whole-genomic mutant collections that offer information-rich, inexpensive microscopy screens and knock-in mutants is not yet available for tissue culture like it is in yeast. Bringing these possibilities to the investigation of mammalian cells and multicellular organisms is a major direction in method development. The important approaches are to maximize the effectiveness and streamline the methods of making knock-ins using CRISPR/Cas (Feng et al. 2017; Fueller et al. 2020), and to develop multiplex staining approaches to visualize subcellular protein distribution (Gut et al. 2018).

### 3.2 Super-resolution microscopy

The diffraction limit of visible light-based microscopes does not resolve signals from two objects that are closer to each other than ∼200 nm. This makes it impossible to study the fine structure of many organelles and molecular complexes using conventional FM. Super-resolution microscopy (SRM) techniques developed over the past 20 years can elegantly circumvent the diffraction limit (Jacquemet et al. 2020). The two main types of SRM are targeted and stochastic (Möckl and Moerner 2020). A targeted method such as Stimulated Emission Depletion Microscopy (STED) uses a laser-scanning set-up with an additional donut-shaped beam to inactivate fluorophores around the center of the diffraction-limited area and only detect signal from a very small central region. Stochastic methods such as Stochastic Optical Reconstruction Microscopy (STORM) rely on spontaneous activation of a small number of sparsely distributed fluorophores at one time point (Rust et al. 2006). Each diffraction-limited spot at these conditions is assumed to come from a single fluorophore or target molecule whose position can be precisely determined as the spot center. SRM opens the gateway to study organelles, subdomains and individual protein complexes at the molecular level without a definitive resolution limit.

The advantages of SRM come together with limitations that reduce the flexibility of these methods compared to conventional FM. In most cases SRM requires custom instrumentation that may not be readily available. For example, a STED microscope needs a system that produces patterned beams, and a STORM system would typically need a much more sensitive camera compared to a regular widefield microscope. The reliance on special fluorophore photophysics poses restrictions on tag selection and sample preparation. For instance, STED uses a very strong laser illumination and requires particularly photostable dyes but the high data acquisition rate makes the method good to study living cells (Wang et al. 2019). Stochastic techniques like STORM and Photo-Activation Localization Microscopy (PALM) require dyes or fluorescent proteins that can blink under defined conditions (Möckl and Moerner 2020). For STORM, the sample needs to be fixed and bathed in a buffer with components for a photochemical reaction that induces blinking. Overall, stochastic techniques favour fixed cells and require long acquisition times to record enough fluorophore localizations. Despite these limitations, SRM has opened a new window into organelle biology.

Until recently, mitochondrial cristae were only observed with EM. STED was used to follow cristae dynamics in living cells and discover that they can undergo fusion and fission on time scales of a few seconds (Kondadi et al. 2020; Segawa et al. 2020). The role of different MICOS subunits was dissected using STED in fixed cells (Stephan et al. 2020). These advances open exciting possibilities to study protein distribution at the level of individual cristae. In ER morphology studies, SRM helped to bridge the gap between EM and FM and to move the view of this organelle beyond a binary division of sheets and tubules. STED revealed that along with regular sheets there exist perforated sheets (Schroeder et al. 2018). Furthermore, using antibodies and STORM it was shown that there are two types of tubes: thin ones that only have reticulons; and thicker, flattened ones that are, in fact, mini-sheets (Wang et al. 2022).

The two remarkable examples taking the SRM development beyond its current limits are MINimal fluorescence photon FLUXes microscopy (MINFLUX) and Resolution Enhancement by Sequential Imaging (RESI). MINFLUX is a combination of targeted and stochastic approaches, as it uses a donut-shaped beam with patterned movement that helps to localize individual fluorophores with nanometer precision, and has high speed owing to the small number of photons required. Such combination of speed and precision allowed direct tracking of individual kinesin motor steps within living cells (Deguchi et al. 2023). RESI is a development of the DNA-Points Accumulation for Imaging nanoscale Topography (PAINT) approach which is a stochastic technique (Schnitzbauer et al. 2017). Here, the target molecule is first labeled with an oligonucleotide. Then a complementary oligonucleotide conjugated to a dye is added. Freely diffusing, it is invisible to the camera because it moves too fast. When bound to a target oligonucleotide, it can be visualized. Dynamic binding and un-binding of the fluorescent oligonucleotide to its target creates blinking that can be used for stochastic localization. To distinguish the localizations that occur next to each other and achieve angstrom-level precision, RESI uses different types of oligonucleotides that act like barcodes for individual target molecules. In this way, distances as short as between neighboring DNA base pairs were resolved and the topology of immune cell plasma membrane receptors was mapped in high detail (Reinhardt et al. 2023). These two examples underscore that the possibilities of modern SRM techniques are no longer constrained by fluorophore localization precision. Now, the main obstacle blocking the ubiquitous application of SRM is the quality of target structure labeling. The three important parameters here are labeling efficiency, label footprint, and signal-to-background ratio. Labeling efficiency is the ratio of target molecules bound to an active fluorophore. Label footprint is defined by the average distance from the target molecule to the bound fluorophore, also called linkage error. Signal-to-background ratio depends on the presence of autofluorescent compounds around the target structure and on the level of unspecific fluorophore conjugation to non-targets.

**Figure 3:**
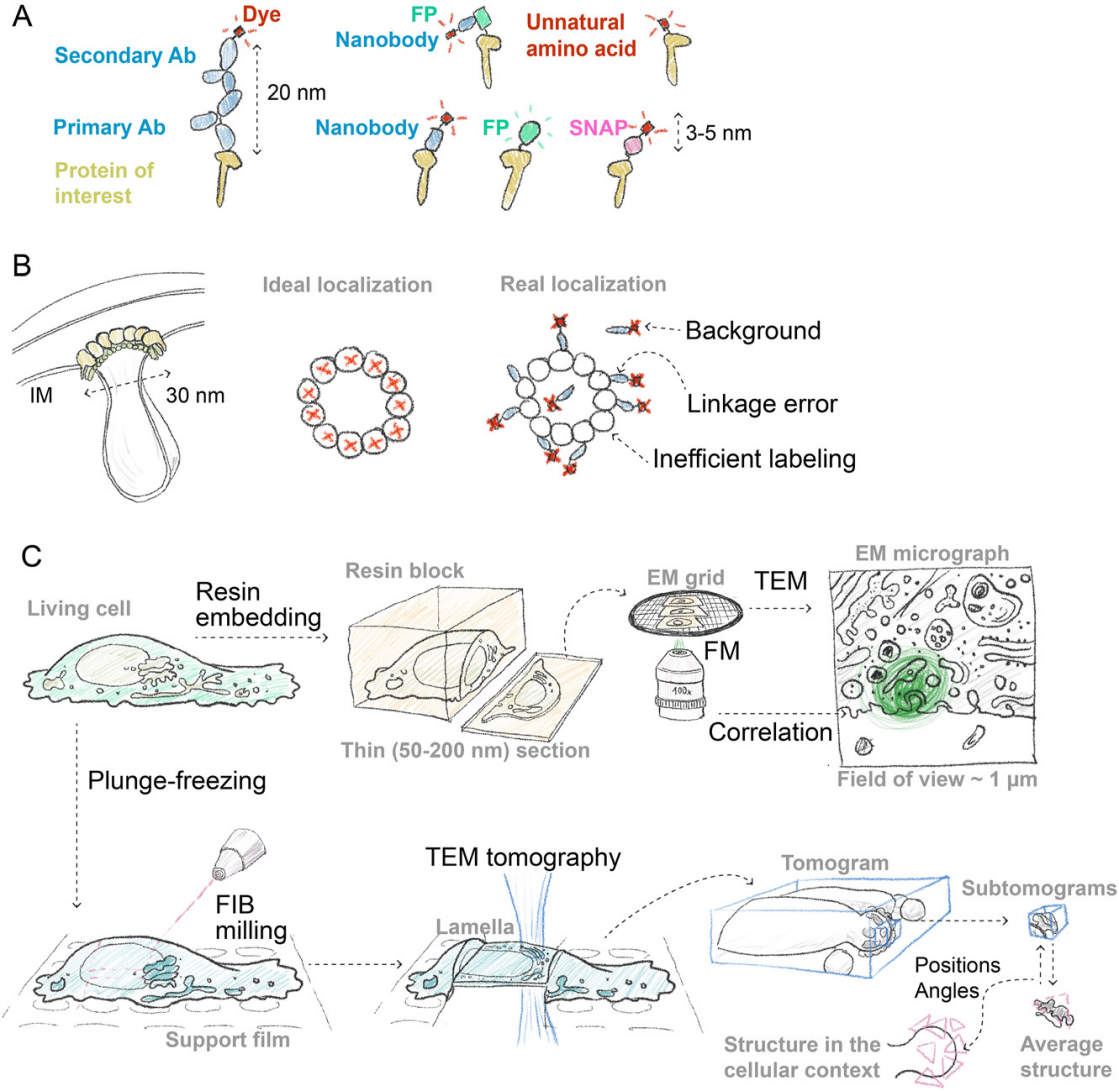
The challenges of visualizing small organelle subdomains and protein complexes by microscopy. (A) The common strategies to label proteins for super resolution microscopy (SRM). The protein of interest can be labeled with: primary and secondary antibodies (Ab); nanobodies derived from camelids; genetically encoded tags such as fluorescent proteins (FPs) and SNAP^®^-tag which can reduce the linkage error to 3–5 nm; combining an anti-GFP nanobody and a GFP tag which increases linkage error but provides brightness boost; incorporating dyes directly using unnatural amino acids, which is a promising approach to reduce linkage error. (B) Left: a hypothetical cut-through model of the mitochondria cristae-organizing complex (MICOS) that shapes the inner membrane of the mitochondria (IM) and stabilizes cristae junctions where MIC60 is shown as the largest protein (olive). Middle: An ideal SRM experiment where every subunit of the hypothetical molecular complex similar to MICOS is precisely localized. Right: a schematic of a more realistic SRM experiment where fluorophore localization precision is perfect, but linkage error, labeling inefficiency, and background staining are added. (C) For electron microscopy (EM) living cells (top left) need to be either fixed and embedded in a plastic block (top row) or frozen (bottom row). Resin-embedded cells are sectioned to produce thin slices and applied to a sample carrier (EM grid). For pre-embedding correlative light and EM (CLEM), the grid is to be imaged directly by fluorescent microscopy (FM) before transmission EM (TEM) and the resulting fluorescence signal later overlayed with an EM micrograph (FM and EM data shown to scale). Plunge-frozen cells grown directly on an EM grid support film are milled by focused ion beam (FIB) to produce lamellas later imaged by TEM tomography. To obtain the structures of large molecules, small portions of the tomogram (subtomograms) are extracted, aligned to an average and used to make a better average structure. The position of each molecule of interest is determined and the average structure (pink triangle) can be placed back into the tomographic volume.

The labeling footprint and efficiency depend on whether antibodies or genetically encoded tagging is used to couple fluorophores to target molecules (Budiarta et al. 2024). Antibody staining is the classic approach to visualize native macromolecules in the cellular context (Figure 3A). The advantage is that it may offer high specificity and low backgrounds, and many good antibodies are available. However the conventional way combines primary and secondary antibodies, with each molecule being around 10 nm. This makes the labeling footprint much larger than the localization precision of many advanced SRM methods. The large size of antibodies also leads to sterical hindrance that lowers labeling efficiency. Antibodies from camelids that have only one variable domain provide a solution. The variable domain alone can be easily expressed and purified as a so-called ‘nanobody’ (Ries et al. 2012; Virant et al. 2018). Nanobodies conjugated to a dye have a much smaller labeling footprint compared to traditional antibodies. Genetically encoded fluorescent tags are another solution to decrease the footprint and increase labeling efficiency as every target molecule would be translated with a tag attached (Figure 3A). Fluorescent proteins are widely applied versatile tags with a large selection of properties, many suitable for SRM (Chudakov et al. 2010). The disadvantage of fluorescent proteins is relatively modest brightness that reduces localization precision. Genetically encoded ‘chemical tags’ such as SNAP-tag^®^ and Halo-tag^®^, which are proteins that specifically bind a bright dye, have advanced labeling for SRM and are widely applied (Saimi and Chen 2023). A popular approach combining genetic and antibody tags is to use a GFP nanobody conjugated to a bright dye to label GFP-tagged proteins (Ries et al. 2012). Such staining is readily applicable to many existing samples containing GFP and retains a relatively small labeling footprint and low background (Figure 3A).

Correct choice and thorough optimization of the labeling to reduce the footprint and background and increase efficiency defines the success of an SRM experiment. Modeling shows that increased footprint and decreased labeling efficiency can make a target structure indiscernible even when localization precision is high (Möckl and Moerner 2020) effectively reducing the system’s resolution (Figure 3B). Fully harnessing the potential of SRM in all fields of cell biology will require incorporating bright fluorophores in a site-specific manner with high specificity. Exciting areas of method development in these directions include the use of unnatural amino-acids and bio-orthogonal chemistry, and developing peptide tags that can be conjugated to a dye enzymatically (Saimi and Chen 2023).

Most SRM methods also require sophisticated data analysis pipelines. This reduces method applicability but helps to compensate for technical and labeling limitations as computational approaches improve. For example, sparse labeling can hinder interpretation of the shape of the observed structure. To study protein complexes with defined architecture or symmetry, a single particle averaging approach can be borrowed from EM (Mendes et al. 2022). In this way, many noisy reconstructions of the same complex taken from the same angle can be averaged to reduce noise and accumulate signal. This approach was successfully applied to SRM of nuclear pores and endocytic machinery (Mund et al. 2018; Szymborska et al. 2013). Heterogeneous assemblies that do not have a defined structure cannot be analyzed by averaging and require analyses based on clustering algorithms (Khater et al. 2020). This approach was applied to imaging MICOS using MINFLUX. While localization precision of individual fluorophores was very high, antibody labeling efficiency did not allow observation of complete MICOS structures at cristae junctions (Pape et al. 2020). To perform a quantitative analysis, the authors developed a new clustering algorithm to group high-precision 3D localizations of the human MIC60 protein and isolate groups that might represent one cristae junction. The comparison of the group shapes with different models of MICOS complex showed a good agreement with a ring-shaped assembly of MIC60 around a 25 nm-wide junction neck.

One key advance over recent years is the introduction of artificial intelligence at different stages of the SRM workflow. Deep learning now offers many more possibilities to process data and detect structures (von Chamier et al. 2021). For example, neural-network-based de-noising was applied to STED imaging of live cell ER. It helped reduce illumination intensity 70 times and collect a dataset that combined spatial resolution with a high acquisition rate of 600 ms per frame and a long imaging duration of about 7 h (Rahm et al. 2024). The further development of efficient labeling and data processing techniques will help to apply SRM to a wider range of problems in organelle biology.

### 3.3 Correlative and traditional EM

Another way to overcome the light diffraction limit is to use electrons for imaging. Their wavelength does not limit the resolution but other properties of these particles restrict the specimen that an electron microscope can handle. A typical sample for a transmission electron microscope must be thinner than most cells (50–500 nm) and free of liquid water due to the high vacuum that is maintained inside of the instrument. This precludes live cell imaging and requires specialized sample preparation techniques aimed at liquid water removal and thinning. The two main approaches for water removal are chemical fixation followed by resin embedding or freezing (covered in Section 3.4). Both frozen and resin-embedded samples can be thinned by a microtome or an additional focused ion beam (FIB) microscope (Figure 3C).

Resin embedding is a classic way to prepare cells and tissues for EM. Hard and elastic, resin helps to produce very thin sections. During or after embedding, samples are contrasted with heavy metal salts that non-specifically bind large protein complexes and membranes. Such staining compensates for poor deflection of electrons by biological macromolecules. The result is a complete overview of cellular ultrastructure that was first attained by transmission EM (TEM) in the middle of the 20th century and defined how we think about organelle shapes and interactions (Palade 1953; Palade and Porter 1954). This view was further enhanced by the introduction of automated image acquisition and processing that led to the development of electron tomography (ET). For ET, a slightly thicker sample is imaged by transmission EM from different angles. The resulting images are used for computational reconstruction of a 3D volume with high axial resolution providing a very detailed view of cellular compartments (Marsh et al. 2001).

The ability to generate an overview of cellular ultrastructure is the main feature of EM but also its primary limitation. EM images are inherently ‘black and white’, and localizing individual proteins in their context is a challenge. A classic approach to add specificity to EM is to stain the sample with antibodies conjugated to gold particles. Such labeling is often very sparse and unspecific because of the limited amount of antigen available for binding on the section’s surface. Another approach is correlative light and electron microscopy (CLEM) that combines the resolution of EM and specificity of FM. CLEM comprises a wide range of protocols and devices to apply EM and FM to the same sample (Scher and Avinoam 2021). The two main varieties are pre-embedding and post-embedding. In pre-embedding CLEM, FM is performed on living or a lightly fixed sample. Then the sample is prepared for EM using one of the standard protocols such as fixation with glutaraldehyde and embedding into epoxy resin with osmium tetroxide contrasting. There are several approaches to relocate the region imaged by FM within the EM sample, typically, using a coordinate system or markings visible in both modalities (Spiegelhalter et al. 2014; Karreman et al. 2016). Post-embedding CLEM usually uses a modified EM sample preparation method that preserves fluorescent protein or dye fluorescence by switching to a more hydrophilic acrylate resin and omitting osmium staining. The sections mounted on the EM specimen carrier (grid) are imaged directly by FM and then by EM (Kukulski et al. 2012b). This approach can be used with most fluorophores and is highly sensitive (Figure 3C, top row).

One of the most powerful applications of CLEM is the ability to match fine membrane ultrastructure with proteins that regulate the shape, composition, and dynamics of organelles. Post-embedding CLEM was applied to correlate the sequence of regulatory protein recruitment to membrane bending and scission occurring during yeast endocytosis (Kukulski et al. 2012a). The view of exactly the same structure obtained by FM and ET revealed that clathrin recruitment does not induce membrane bending, which only starts after actin polymerization at the endocytic site. ET has the highest isotropic resolution but usually provides very small fields of view. When a larger field of view is required, FIB-thinning combined with scanning electron microscopy (SEM) can be applied. This approach, also termed ‘slice and view’, relies on an integrated FIB-SEM instrument. The sample surface is imaged by SEM, then a thin layer on the surface is removed by FIB, and the new surface is imaged again. These cycles can be used to obtain very large 3D views of cells and tissues (Heinrich et al. 2021; Xu et al. 2017). The best possible 3D resolution of FIB-SEM datasets is usually 2–5 times lower than that of ET of plastic-embedded specimens. Still, this is enough to visualize organelles and small transport vesicles. Pre-embedding CLEM combined with FIB-SEM revealed the morphology and composition of complex vesicular-tubular membrane trafficking intermediates in between the ER and the Golgi apparatus and in the *trans-*Golgi network (TGN) (Stockhammer et al. 2024a; Weigel et al. 2021).

The major limitation of CLEM is the resolution gap between the EM and FM imaging modalities. A group of different structures visible by EM can hide behind one diffraction-limited spot in FM imaging (Figure 3C, top row). To overcome this, several works reported ways to combine SRM with CLEM to assign target molecule localization to EM structures with more precision in post-embedding CLEM protocols. The problem is that resin embedding significantly changes the fluorophore environment that can hinder the photophysical properties required for blinking. The solutions proposed to use conventional and specifically designed photoswitchable proteins, or changing the resin embedding protocol (Franek et al. 2024; Fu et al. 2020; Peddie et al. 2017). The development and wide adoption of new labeling techniques (see Section 3.2) opens exciting possibilities to advance SRM CLEM workflows to get the best of both EM and light microscopy from one sample.

### 3.4 *In situ* cryogenic ET (cryo-ET)

Resin-embedded samples are easy to work with and reveal a great deal of organellar ultrastructure. However, the internal native macromolecular structures are denatured due to the chemical treatments. Instead, biological molecules can be fully preserved by freezing – an alternative way to prepare a sample without liquid water. To retain native cellular structure, freezing needs to be carried out in a way that prevents formation of ice crystals that damage it. One way is to freeze the sample by plunging it into liquid ethane (Adrian et al. 1984). To ensure quick heat transfer, the specimen cannot be thicker than 10 µm. Another way is to use a machine that applies liquid nitrogen to the specimen under a very high pressure (high-pressure freezer) that inhibits ice crystal formation. This method can handle specimens up to a few hundred micrometers, even small organisms and pieces of tissue. The resulting samples contain all biological molecules immobilized in amorphous (vitreous) ice that preserves them in native-like conformations with high-resolution details intact. After preparation, the specimens are constantly maintained at cryogenic temperatures (Figure 3C, bottom row). Usually, the frozen samples are still too thick to be imaged by TEM, and therefore need to be thinned down by making sections or using a FIB microscope.

The traditional method of ultramicrotomy was adapted to make thin sections directly from cryogenic samples. It provided one of the views of native cellular ultrastructure non-hindered by chemical fixation (Al-Amoudi et al. 2004). However, cryogenic sections suffer from knife-damage artifacts and the sections are hard to handle and attach to EM grids. The alternative method to obtain thin cellular regions for TEM is to mill away material by a cryogenic FIB-SEM instrument to produce 100–200 nm-thick slabs (lamellas) of vitreous material (Rigort et al. 2012). The ultrastructure inside the lamellas remains intact with the damage concentrated on the surfaces (Figure 3C, bottom row). Sections or lamellas of the frozen samples can then be imaged by ET to obtain a 3D view of the cell with isotropic resolution (Asano et al. 2016; Mahamid et al. 2016). The relatively new field of *in-situ* cryo-ET has now rendered an unprecedented view into native organization of unicellular and small multicellular organisms such as *Caenorhabditis elegans* (Asano et al. 2015; Berger et al. 2023; Schiøtz et al. 2024; Shimakawa et al. 2024; Wietrzynski et al. 2020).

Vitrified sections or lamellas of cells contain all the biological molecules in their near-native state but only few of them can be directly observed. The major limitation of *in-situ* cryo-ET comes from the extremely high sensitivity of the frozen samples to electron damage. The electron doses used for cryogenic microscopy are thus very low making signal to noise ratio very high. In this way, only relatively low resolution features such as positions of membranes and shapes of large complexes such as ribosomes are clearly visible in the tomographic reconstructions because they create enough signal (Figure 3C, bottom row). The high-resolution information is also present in the reconstructions but is buried under the noise. The same problem hinders regular cryogenic EM that is used to determine structures of purified molecular complexes by single particle analysis. The solution is to average similar noisy particles to decrease the noise and bring up the high-resolution information (Frank 2018). The averaging approach can be combined with ET to derive molecular structures directly from intact cells. In this way, small portions of the initial tomographic reconstruction that contain the molecule of interest are cropped and compared to a reference structure. Their orientations are determined and used to average the subtomograms and produce a better reference structure with high resolution (Wan and Briggs 2016). Together with the average structure, this approach produces an additional important piece of information: how the molecules of interest are positioned in the cellular context. This gives a unique opportunity to investigate the molecular organization of the cell and connect structure with ultrastructure (Figure 3C, bottom row). Technical and data processing developments helped to move the resolution of subtomograms close to that of single particle analysis (Tegunov et al. 2021). The possibility of visualizing the positions of individual macromolecules in the original tomogram helped to determine how the major secretory pathway membrane coat complexes clathrin, COPII, COPI, and retromer organize their cargo and induce vesicle budding *in vitro* and *in vivo* (Bykov et al. 2017; Dodonova et al. 2015; Kovtun et al. 2018, 2020; Pyle et al. 2025). The method was also useful in investigating the organization of mitochondrial electron transport chain revealing the role of ATP synthase in maintaining cristae shape in different eukaryotic supergroups (Davies et al. 2012; Mühleip et al. 2021). In *Chlamydomonas* mitochondria, subtomogram averaging was used to visualize the respiratory chain supercomplexes that were only previously ever observed in isolation (Waltz et al. 2025). On the individual protein complex level, subtomogram averaging was most successfully applied to determine ribosome structures in different translational states in the cytosol, next to ER membranes, and under stress (Fedry et al. 2024; Gemmer et al. 2023; Xing et al. 2023; Xue et al. 2022). The prevalence of high resolution ribosome structures compared to smaller complexes points to the main limitation of subtomogram averaging: small molecules are much harder to average because their noisy images do not contain large features used to compare and determine subtomogram orientation. As with conventional EM, *in situ* cryo-ET can resolve the overall ultrastructure and large complexes but lacks specificity for many other molecules.

Molecular specificity can be added to *in situ* cryo-ET with cryogenic CLEM (cryo-CLEM). The sample can be visualized before or after thinning using FM setups with cooled stages or integrated FM/EM devices (Pierson et al. 2024). FM imaging can help to find a specific cell to guide FIB-milling or to pinpoint a specific structure within the cell (Arnold et al. 2016). It was recently used to determine the structure and organization of the yeast ER-mitochondria encounter structure providing the first high-resolution glimpse into the organization of this membrane domain (Wozny et al. 2023). The advantages of cryo-CLEM are its relative simplicity and low cost of instrumentation compared to all other expenses in cryo-EM. Another advantage is that fluorophore bleaching is almost absent at cryogenic temperatures giving a possibility to collect more signal, multiple z-stacks, and to use fluorescence-based autofocus (Kaufmann et al. 2014; Schorb et al. 2017). The limitations of cryo-CLEM come with the inability to use objectives with high numerical aperture. This lowers the resolution of cryo-FM data and further widens the gap between FM and EM.

Bridging the resolution gap is an active area of method development (Dahlberg and Moerner 2021; Möckl and Moerner 2020; Moser et al. 2019; Wolff et al. 2016; Zanetti-Domingues et al. 2024). One of the main directions is introducing SRM into cryo-CLEM. Since the blinking mechanisms commonly used in regular SRM stop working at cryogenic conditions, cryo-CLEM requires developing new fluorophores or new ways to make the old ones blink at cryo-temperatures. Another approach to detect molecules that are too small for cryo-ET is to use EM-compatible tags that are large enough to be found by subtomogram analysis, or have high electron density due to metal content (Fung et al. 2023; Kikkawa and Yanagisawa 2022; Silvester and Baker 2024; Yao et al. 2025). The developments in artificial intelligence-based protein design (Kortemme 2024) hold a great promise on making specific ‘shape-tags’ for cryo-ET that will be efficient and biocompatible (Hsia et al. 2021; Ingraham et al. 2023; Kortemme 2024; Meador et al. 2024). The development of data processing tools for cryo-ET aims to extract higher resolution information with less data and get insights from the cellular context analysis. The key advances which have already made a great difference are: increasing the data collection speed by new acquisition schemes (Eisenstein et al. 2023), denoising raw tomographic reconstructions to see more detail by eye (Buchholz et al. 2019), and refining reconstructions to calculate high-resolution average structures (Ni et al. 2022; Tegunov et al. 2021; Zivanov et al. 2022). The new exciting development directions are: using machine learning for unbiased mining of the dataset for different particles (de Teresa-Trueba et al. 2023), improving membrane segmentation and morphometry with neural networks (Grotjahn 2025; Lamm et al. 2022), and detecting multiple conformations of molecular complexes (Rangan et al. 2024). The final goal is to integrate high-resolution cryo-ET data with other modalities to obtain a complete, dynamic picture of cellular compartments (CLEM and cryo-ET reviewed in more detail in (McCafferty et al. 2024)).

## 4 Perspectives

The developments of proteomic and imaging methods helped to update our picture of cellular organelles. Instead of a general map of organelles as separate continents in the ocean of cytosol, we have started to get a more nuanced and detailed view. Now we see that there are many different ways how proteins can organize into suborganellar structures with various sizes and complexity (Figure 1). Similarly to new cartography methods that produced more detailed or specialized atlases of the continents, cities and habitats, proteomics and imaging accumulated vast amounts of data on protein distributions, interactions, and movements within and between organelles. The future challenge of cell biology is integrating these datasets to describe the full diversity of organelles and their subdomains in different cell types. Similarly to online maps that allow us to traverse different scales of geography and visualize the most interesting aspects, integrating imaging and proteomics will give us a dynamic, holistic cellular atlas.
